# Clinical Presentations and Surgical Outcomes in Patients With Nontraumatic Acute Abdominal Pain: A Prospective Analysis

**DOI:** 10.7759/cureus.75772

**Published:** 2024-12-15

**Authors:** Rajat Sharma, Siddharth B Lonare, Savijot Singh, Hamza Al-Dwlai, Rajeev Ranjan

**Affiliations:** 1 General Surgery, Maharishi Markandeshwar Medical College and Hospital (MMMCH), Solan, IND; 2 General Surgery, Byramjee Jeejeebhoy (BJ) Government Medical College and Sassoon General Hospital, Pune, IND; 3 General Surgery, Adesh Medical College and Hospital, Kurukshetra, IND; 4 General Surgery, Luton and Dunstable University Hospital, Luton, GBR; 5 General Surgery, Netaji Subhas Medical College and Hospital, Patna, IND

**Keywords:** acute abdominal pain, complications, nontraumatic, patient outcomes, surgical management

## Abstract

Background: Nontraumatic acute abdominal pain is a common presentation in emergency settings, often requiring surgical intervention. This study aimed to explore the clinical presentations, surgical management, and outcomes in patients with nontraumatic acute abdominal pain, providing insights for improving management strategies.

Methods: This observational study was conducted at a tertiary care hospital in North India and included 433 patients who underwent elective and emergency abdominal surgeries from June 2021 to May 2023. Data were collected on patient demographics, comorbidities, duration of symptoms, initial presenting symptoms, and surgical procedures performed. Laboratory parameters were assessed preoperatively, and postoperative outcomes, including complications, recovery metrics, and length of hospital stay, were recorded. Statistical analyses, including logistic regression, were utilized to determine the adjusted odds ratios for significant predictors of complications.

Results: A total of 433 patients were included in the analysis, with a mean age of 42.3 ± 12.1 years. The study revealed that 5.5% of patients experienced complications, with wound infections (3.0%) and intra-abdominal abscesses (0.9%) being the most common. Recovery metrics indicated that the mean time to resume oral intake was 2.5 ± 1.2 days, the time to first bowel movement was 3.0 ± 1.5 days, and the duration of the postoperative hospital stay averaged 5.0 ± 2.0 days. Logistic regression analysis identified significant predictors of complications, including diabetes mellitus (adjusted OR, 2.02; p < 0.001), hypertension (adjusted OR, 1.44; p = 0.025), and intraoperative findings such as appendiceal perforation (adjusted OR, 2.14; p < 0.001).

Conclusion: The study underscores the critical role of timely diagnosis and appropriate surgical management in patients with nontraumatic acute abdominal pain. Recognizing high-risk factors, such as diabetes and elevated American Society of Anesthesiologists scores, can enhance surgical decision-making and improve patient outcomes. These findings advocate for refined management protocols and a multidisciplinary approach to optimize care for patients presenting with acute abdominal pain.

## Introduction

Nontraumatic acute abdominal pain is a critical clinical challenge, representing 10%-20% of emergency department visits worldwide [1]. This symptom encompasses a wide spectrum of etiologies, ranging from self-limiting conditions to life-threatening emergencies, necessitating prompt diagnosis and management to avert severe complications. Common causes include acute appendicitis, acute cholecystitis, small bowel obstruction (SBO), pancreatitis, and perforated peptic ulcer disease, each posing distinct diagnostic and therapeutic challenges.

Acute appendicitis, the most frequent nontraumatic surgical emergency, has a global incidence of 86-106 cases per 100,000 people annually, particularly affecting young adults [2]. Similarly, gallstone-related complications like acute cholecystitis, prevalent in 10%-15% of adults, often necessitate urgent surgical intervention, especially in older patients or those with diabetes [3]. SBO, responsible for 15% of hospital admissions due to abdominal pain, arises primarily from adhesions, hernias, or malignancies, requiring precise surgical timing to prevent bowel ischemia [4]. Pancreatitis, accounting for 3%-5% of cases, presents a variable severity spectrum, often requiring intensive care or surgical intervention in severe forms [5]. Meanwhile, perforated peptic ulcers, frequently linked to *Helicobacter pylori* infection and nonsteroidal anti-inflammatory drug use, pose a diagnostic challenge, particularly in resource-constrained settings [6].

Advancements in imaging, notably computed tomography (CT) and ultrasound (USG), have enhanced diagnostic precision, with accuracies exceeding 90% for conditions such as appendicitis and SBO [7]. However, gaps persist in translating these advancements into optimal clinical outcomes. Timely surgical decision-making remains challenging, particularly in ambiguous cases where clinical signs, imaging findings, and laboratory markers like elevated white blood cell count (WBC) and C-reactive protein (CRP) are inconclusive. Furthermore, previous studies have primarily focused on individual conditions, leaving a gap in comprehensive assessments of varied presentations and their impact on surgical outcomes in diverse populations [8].

This study aimed to fill this gap by systematically evaluating the clinical presentations, diagnostic approaches, and surgical outcomes in patients presenting with nontraumatic acute abdominal pain. By integrating clinical, laboratory, and imaging data, this research seeks to identify patterns that facilitate timely decision-making, reduce diagnostic delays, and improve outcomes in emergency care settings. Such insights are critical to bridging the divide between diagnostic advancements and their practical implementation, ultimately enhancing patient care and resource utilization.

## Materials and methods

Study design and setting

This study was a prospective, observational analysis conducted in the Department of General Surgery at a tertiary care center in North India. The study period spanned from June 2021 to May 2023. This setting allowed for a comprehensive evaluation of diverse nontraumatic abdominal emergencies across various demographics.

Study population

The study included adult patients presenting to the emergency department with acute abdominal pain of nontraumatic origin. Eligible patients were aged 18 years or older, with pain onset within the past 48 hours and no history of trauma or external abdominal injury. Exclusion criteria comprised a prior history of abdominal surgery, chronic abdominal pain persisting for more than 48 hours or with a recurrent pattern, and any recent trauma to the abdomen. This approach ensured a focus on acute, nontraumatic causes of abdominal pain in the study population.

Sampling technique

The study utilized a consecutive sampling technique, where all eligible adult patients presenting to the emergency department with acute abdominal pain of nontraumatic origin during the study period were included. Consecutive sampling ensured that no eligible patient was excluded, providing a representative sample of the population and reducing selection bias.

Sample size calculation

A minimum sample size of 433 patients was calculated using power analysis to detect clinically meaningful differences in presentation patterns and operative outcomes. The analysis aimed for a statistical power of 80% and a significance level of 5%, which are standard thresholds in clinical research. Based on previous studies evaluating nontraumatic acute abdominal pain, an effect size of 0.5 was assumed, reflecting moderate clinical relevance in the anticipated differences between diagnostic outcomes and surgical interventions [9,10].

Data collection

The questionnaire was validated through a structured process to ensure its reliability and appropriateness. Initially, face and content validity were established by consulting a panel of five experts, including senior surgeons and emergency medicine specialists, who reviewed the questionnaire for relevance, clarity, and comprehensiveness. Their feedback was incorporated into the final version. A pilot study was conducted with 30 patients meeting the inclusion criteria to assess feasibility and comprehension. Internal consistency was evaluated using Cronbach's alpha, achieving a value of 0.82, indicating good reliability. Additionally, test-retest reliability was assessed by administering the questionnaire to 10 patients twice, two weeks apart, yielding a strong intraclass correlation coefficient of 0.88.

Data collection involved three main components. The first component was clinical evaluation, which included a detailed recording of the patients' pain characteristics, such as location, onset, type, and intensity on a numeric pain scale, along with associated symptoms like nausea, vomiting, fever, anorexia, and changes in bowel or urinary habits. Physical examination findings, including abdominal tenderness, rebound tenderness, guarding, rigidity, bowel sounds, and the presence of any mass, were systematically assessed and documented.

The second component focused on laboratory investigations; blood samples were obtained upon admission to perform a complete blood count, liver function tests, renal function tests, CRP, serum amylase and lipase, and lactate levels, with abnormal values noted for diagnostic insights and preoperative assessment. In cases suspected of infection or inflammation, additional markers, such as procalcitonin levels, were recorded when available.

The third component encompassed imaging and radiological assessment. All patients underwent an initial abdominal USG performed by an experienced radiologist, which was essential for evaluating gallbladder disease, appendicitis, or free fluid in cases of suspected perforation. Patients with inconclusive USG findings or high clinical suspicion for conditions such as bowel obstruction, ischemia, or perforation were subsequently referred for contrast-enhanced CT scans, which were instrumental in detecting complex or life-threatening conditions requiring urgent surgical intervention.

Operative management and intervention

Patients with a definitive surgical diagnosis were categorized according to the type of pathology and the urgency of the required intervention. Operative details were systematically recorded to ensure comprehensive documentation. The type of surgery performed included procedures such as laparoscopic or open appendectomy, cholecystectomy, bowel resection, adhesiolysis, or other relevant surgical interventions tailored to the patient's specific condition. Intraoperative findings were meticulously documented, highlighting critical observations such as appendiceal perforation, gangrenous bowel segments, or the presence of abscess formation, which informed postoperative management strategies. Following surgery, a standard postoperative protocol was implemented, encompassing effective pain management, meticulous wound care, and vigilant monitoring for signs of infection or potential complications, including ileus, sepsis, or anastomotic leak.

Outcome measures and follow-up

Patients were followed postoperatively for a minimum of 30 days to assess various outcomes related to their surgical intervention. The primary outcome measured was the hospital stay duration, which included the total length of stay from the time of admission to discharge. In addition, postoperative complications were carefully documented, focusing on specific events such as wound infections, intra-abdominal abscess formation, respiratory complications, and rates of readmission within the follow-up period. To evaluate functional recovery, we tracked recovery metrics, including the time taken for patients to resume oral intake, the return of bowel function, and the ability to mobilize independently.

Statistical analysis

All data were analyzed using SPSS version 25.0 (IBM Corp., Armonk, NY) to ensure rigorous statistical evaluation. Continuous variables were expressed as mean ± standard deviation, while categorical data were summarized as frequencies and percentages for clarity. Logistic regression was used to identify potential confounders, such as age, comorbidities, and clinical predictors influencing postoperative outcomes. A p value of <0.05 was considered statistically significant.

Ethical considerations

The study received ethical clearance from the Institutional Ethics Committee (approval number: MMMCH/IEC/21/559). Informed consent was obtained from all participants prior to inclusion, ensuring adherence to the ethical principles outlined in the Declaration of Helsinki. Data confidentiality and participant privacy were strictly maintained throughout the study.

## Results

The study included 433 patients with a mean age of 42.3 years and a mean body mass index of 24.7 kg/m^2^. The gender distribution was skewed toward men (60.3%). Comorbid conditions were common, with 21.2% having diabetes, 18.0% with hypertension, and 8.5% with cardiovascular disease. Chronic respiratory conditions affected 5.5% of the cohort, and 3.9% had renal disease. An additional 5.3% reported other comorbidities (Table 1).

**Table 1:** Demographic and clinical characteristics of study participants (N = 433) SD: standard deviation

Characteristic	Frequency/mean ± SD	%
Age (years)	42.3 ± 12.1	-
Gender		
Male	261	60.3
Female	172	39.7
Body mass index (kg/m^2^)	24.7 ± 3.2	-
Comorbidities		
Diabetes	92	21.2
Hypertension	78	18.0
Cardiovascular disease	37	8.5
Chronic respiratory disease	24	5.5
Renal disease	17	3.9
Other	23	5.3

Intraoperative observations showed that 18.9% of patients had appendiceal perforation, and 10.4% had gangrenous bowel. Abscess formation occurred in 7.2% of cases, while intestinal obstruction and perforated viscus were noted in 8.3% and 4.2% of patients, respectively. Other findings were present in 1.8% of the cohort (Table 2).

**Table 2:** Clinical presentation, surgical procedures, and intraoperative findings in patients with acute abdominal pain (N = 433) SD: standard deviation

Variables	Frequency/mean ± SD	%
Duration of symptoms (days)	24.6 ± 10.2	-
Initial presenting symptoms		
Nausea	182	42.0
Vomiting	147	33.9
Fever	134	30.9
Anorexia	99	22.9
Bowel changes	89	20.6
Urinary changes	42	9.7
Surgical procedure		
Laparoscopic appendectomy	122	28.2
Open appendectomy	63	14.5
Laparoscopic cholecystectomy	89	20.6
Open cholecystectomy	28	6.5
Bowel resection	76	17.6
Adhesiolysis	26	6.0
Hernia repair	21	4.8
Other procedures	3	0.7
Finding		
Appendiceal perforation	82	18.9
Gangrenous bowel	45	10.4
Abscess formation	31	7.2
Intestinal obstruction	36	8.3
Perforated viscus	18	4.2
Other	8	1.8

Laboratory tests indicated that 14.1% of patients had low hemoglobin levels, while elevated WBC counts and CRP were seen in 23.8% and 28.2%, respectively, suggesting inflammation. Elevated serum amylase and lipase were noted in 10.2% and 8.3% of patients, hinting at possible pancreatic involvement. Imaging findings showed cholelithiasis in 11.8%, appendicitis in 18.9%, and free fluid in 8.3% on USG. CT scans revealed bowel obstruction (10.9%), perforation (7.2%), and intra-abdominal abscesses (5.3%) (Table 3).

**Table 3:** Laboratory and imaging findings in patients with acute abdominal pain (N = 433) SD: standard deviation; CRP: C-reactive protein; USG: ultrasound; CT: computed tomography

Parameter	Frequency (%)	Mean ± SD
	Abnormal range	
Hemoglobin (g/dL)	61 (14.1%)	12.5 ± 2.1
White blood cell count (×10^3^/L)	103 (23.8%)	9.8 ± 4.3 × 10³
CRP (mg/L)	122 (28.2%)	15.8 ± 10.2
Serum amylase (U/L)	44 (10.2%)	91.09 ± 33.07
Serum lipase (U/L)	36 (8.3%)	75.08 ± 31.56
USG findings		
Cholelithiasis	51 (11.8%)	-
Appendicitis	82 (18.9%)	-
Free fluid	36 (8.3%)	-
CT findings		
Bowel obstruction	47 (10.9%)	-
Perforation	31 (7.2%)	-
Intra-abdominal abscess	23 (5.3%)	-

Complications were observed in 5.5% of patients, with wound infections (3.0%) and intra-abdominal abscesses (0.9%) being the most common. Other complications included respiratory complications (0.7%) and urinary tract infections (0.5%). Readmissions and deep vein thrombosis occurred in 0.2% each. Patients resumed oral intake within an average of 2.5 days, bowel movements in 3.0 days, and ambulation in 1.8 days. The average hospital stay was 5.0 days (Table 4).

**Table 4:** Postoperative complications and recovery metrics in patients undergoing surgery for acute abdominal pain (N = 433) SD: standard deviation

Variables	Frequency/mean ± SD	%
Complication (n = 24)		
Wound infection	13	3.0
Intra-abdominal abscess	4	0.9
Respiratory complications	3	0.7
Urinary tract infection	2	0.5
Deep vein thrombosis	1	0.2
Readmission	1	0.2
Recovery metric		
Time to resume oral intake (days)	2.5 ± 1.2	-
Time to first bowel movement (days)	3.0 ± 1.5	-
Time to ambulation (days)	1.8 ± 0.9	-
Duration of postoperative hospital stay (days)	5.0 ± 2.0	-
Time to discharge (days)	5.5 ± 2.2	-

Age, diabetes, and hypertension were linked to higher complication risks (odds ratios, ORs: 1.13, 2.02, and 1.44, respectively). A symptom duration >24 hours increased risk (OR = 1.76). Laparoscopic surgery had lower complication odds (OR = 0.68). Intraoperative findings of appendiceal perforation (OR = 2.14) and bowel gangrene (OR = 3.25) were significant, as were higher American Society of Anesthesiologists (ASA) scores (OR = 2.54). Elevated CRP, low albumin, and high lactate levels were also predictors (ORs: 1.67, 1.85, and 2.03) (Table 5).

**Table 5:** Adjusted odds ratios for predictors of adverse surgical outcomes (N = 433) ASA: American Society of Anesthesiologists; CRP: C-reactive protein

Predictor variable	Adjusted odds ratio	95% confidence interval	p value
Age (per 10-year increase)	1.13	1.11-1.62	0.002
Male	1.15	0.86-1.57	0.309
Diabetes mellitus	2.02	1.65-3.09	<0.001
Hypertension	1.44	1.18-2.16	0.025
Duration of symptoms >24 hours	1.76	1.22-2.44	0.01
Laparoscopic vs. open surgery	0.68	0.48-0.88	0.005
Intraoperative finding			
Appendiceal perforation	2.14	1.44-3.07	<0.001
Bowel gangrene	3.25	2.11-5.19	<0.001
ASA score (III/IV vs. I/II)	2.54	1.77-3.56	<0.001
CRP level >10 mg/L	1.67	1.23-2.19	0.015
Albumin level <3.5 g/dL	1.85	1.31-2.66	0.008
Preoperative lactate >2 mmol/L	2.03	1.46-2.89	0.005

## Discussion

The findings of our study reveal critical insights into the clinical characteristics, recovery metrics, complications, and predictors of outcomes in patients undergoing surgical interventions for acute abdominal conditions. Our cohort had a mean age of 42.3 years, with a higher prevalence of male patients (60.3%), aligning with the studies by Shinde et al. and Cervellin et al., which indicate that men are at greater risk for acute abdominal emergencies due to factors such as lifestyle and health-seeking behaviors [11,12].

The demographic data indicated a significant proportion of patients with comorbidities, including diabetes (21.2%) and hypertension (18.0%). These findings are consistent with those of Shuvo et al., who noted that patients with diabetes are more prone to complications due to impaired wound healing and immune response [13]. Furthermore, the increased odds of adverse outcomes associated with hypertension, reflected in our multivariable analysis (OR, 1.44; 95% confidence interval, CI, 1.18-2.16), echo the findings of Lizano-Díez et al., highlighting the need for careful perioperative management of hypertensive patients [14].

Recovery metrics showed that patients had a mean time to resume oral intake of 2.5 days and an average postoperative hospital stay of 5.0 days. These results are comparable to those of Canzan et al., who emphasized that early mobilization and oral feeding significantly improve recovery times [15]. The reported time to first bowel movement (3.0 days) and ambulation (1.8 days) further illustrate the effectiveness of protocols that promote early recovery, as supported by Jønsson et al. [16].

In terms of complications, wound infections and intra-abdominal abscesses were the most common, affecting 3.0% and 0.9% of patients, respectively. These rates align with Sartelli et al., who found similar surgical site infection rates in surgical populations, underscoring the persistent challenge of managing postoperative infections [17]. Moreover, our study observed a 0.2% readmission rate, which is consistent with findings by Kacprzyk et al., which identified readmission as a significant indicator of surgical quality and patient safety [18].

Our analysis identified several predictors of complications, reinforcing findings from previous research. The significant association between appendiceal perforation (OR, 2.14; 95% CI, 1.44-3.07) and bowel gangrene (OR, 3.25; 95% CI, 2.11-5.19) with adverse outcomes corroborates the conclusions of Zewdu et al. and Kaewlai et al., who indicated that the severity of intraoperative findings directly impacts postoperative morbidity [19,20].

Importantly, our logistic regression analysis revealed that elevated preoperative CRP levels (>10 mg/L) and low serum albumin levels (<3.5 g/dL) were associated with increased odds of complications, with ORs of 1.67 (95% CI, 1.23-2.19) and 1.85 (95% CI, 1.31-2.66), respectively. This finding supports the work of Yamazaki et al., which highlighted the predictive value of inflammatory markers and nutritional status in surgical outcomes [21]. The significant correlation between ASA scores (III/IV vs. I/II) and adverse outcomes (OR, 2.54; 95% CI, 1.77-3.56) underscores the necessity of a thorough preoperative evaluation to optimize patient outcomes, as supported by Bhat et al. [22].

Finally, our data on surgical techniques demonstrated that laparoscopic approaches were associated with lower complication rates (OR, 0.68; 95% CI, 0.48-0.88), aligning with findings from Watrowsk et al. and Kajihara et al., who reported that minimally invasive surgery reduces complications and hospital stays compared to open surgery [23,24].

This study highlights critical insights into the clinical characteristics, recovery trajectories, complications, and predictors of outcomes in patients undergoing surgical interventions for acute nontraumatic abdominal emergencies. The predominance of male patients and the significant impact of comorbidities, such as diabetes and hypertension, emphasize the need for targeted perioperative management. Recovery metrics, including early resumption of oral intake and mobilization, coupled with low complication rates, underscore the effectiveness of current surgical protocols while challenges in infection management persist. Elevated preoperative CRP, low serum albumin, and high ASA scores were identified as significant predictors of adverse outcomes, reinforcing the importance of preoperative risk stratification. Additionally, the lower complication rates and shorter hospital stays associated with laparoscopic techniques validate the role of minimally invasive surgery in optimizing outcomes. These findings provide actionable guidance for improving clinical practices, tailoring patient care, and refining protocols to enhance safety and recovery in acute abdominal conditions.

Limitations

This study has several limitations that warrant consideration. First, its single-center design may constrain the generalizability of the findings to other institutions or regions with differing demographic and clinical profiles. Second, the observational nature of the study introduces potential biases, particularly in patient selection and reporting of outcomes, which could impact the validity of the conclusions. Third, while the sample size was adequate for identifying significant associations, it may have been underpowered to detect less frequent predictors of outcomes. Fourth, reliance on clinical records for preoperative assessments may have resulted in incomplete data capture, especially regarding variables like baseline nutritional status and detailed postoperative recovery metrics. Finally, unmeasured confounding factors, such as variations in surgeon expertise or patient adherence to postoperative care, may have influenced the results, necessitating cautious interpretation.

## Conclusions

This study emphasizes the varied clinical presentations and management of nontraumatic acute abdominal pain, highlighting the critical role of early recognition and timely surgical intervention in enhancing patient outcomes. Our findings reveal common symptoms, including nausea and vomiting, and significant intraoperative findings such as appendiceal perforation and bowel gangrene. Notably, comorbidities like diabetes and hypertension were identified as predictors of postoperative complications, reinforcing the need for careful preoperative evaluation.
